# Health Mindset in Pediatric Inflammatory Bowel Disease: Exploring the Relationship Between Health Mindset and Key Physical and Psychosocial Outcomes

**DOI:** 10.3390/children13050658

**Published:** 2026-05-08

**Authors:** Callie Goodman, Nicole Neiman, Ashley Dunn, Ann Ming Yeh, Rachel Bensen, Claudia Mueller, Anava Wren

**Affiliations:** 1 Division of Gastroenterology, Hepatology and Nutrition, Department of Pediatrics, Stanford University School of Medicine, Stanford, CA 94304, USA; callieg@stanford.edu (C.G.); nicole.neiman@lmunet.edu (N.N.); adunn2@stanford.edu (A.D.); annming@stanford.edu (A.M.Y.); bensen@stanford.edu (R.B.); 2 Department of Psychology, Palo Alto University, Palo Alto, CA 94304, USA; 3 DeBusk College of Osteopathic Medicine, Lincoln Memorial University, Knoxville, TN 37752, USA; 4 Department of Surgery, Stanford University School of Medicine, Stanford, CA 94304, USA; mueller4@stanford.edu

**Keywords:** adolescent and young adults, inflammatory bowel disease, health mindset, mindset

## Abstract

**Highlights:**

**What are the main findings?**

- This is the first study to examine health mindset among adolescents and young adults with inflammatory bowel disease, demonstrating that it could be reliably assessed in this sample.
- The results found that a growth health mindset was cross-sectionally associated with lower pain interference, lower Crohn’s disease activity, and better global health.

**What are the implications of the main findings?**

- Health mindset may be important in explaining variability in adjustment and important health outcomes among adolescents and young adults with inflammatory bowel disease.
- Mindsets have been shown to be modifiable psychological constructs, suggesting that interventions designed to cultivate a growth health mindset may improve physical and psychosocial outcomes among young people with inflammatory bowel disease.

**Abstract:**

**Background/Objectives:** Adolescents and young adults (AYAs) with inflammatory bowel disease (IBD) experience persistent physical symptoms and psychosocial challenges that can impair functioning and quality of life. Health mindset, beliefs about whether health is fixed versus malleable and responsive to effort, is linked to positive health outcomes but has not been examined in AYAs with IBD. This study evaluated the internal reliability of a health mindset measure in AYAs with IBD and examined associations between health mindset and depressive symptoms, peer relationships, global health, pain interference, fatigue, and disease activity. **Methods:** Participants were 101 AYAs with IBD (M = 18.4 years; 54.4% ulcerative colitis; 63.4% female) recruited from an outpatient pediatric IBD clinic and a national IBD network. Participants completed a one-time online survey consisting of the Health Mindset Scale, Patient-Reported Outcomes Measurement Information System (PROMIS) measures for physical, psychosocial, and global health outcomes, and IBD disease activity indices. **Results:** The Health Mindset Scale demonstrated good internal reliability in this sample. Additionally, a growth health mindset was significantly associated with lower pain interference, lower Crohn’s disease activity, and better global health. No significant associations were observed between health mindset and depressive symptoms, peer relationship quality, fatigue, or ulcerative colitis disease activity. **Conclusions:** This novel study provides initial evidence that health mindset is associated with clinically meaningful outcomes in AYAs with IBD. These findings highlight that health mindset may be an important psychological construct in chronic illness adjustment and management, extending mindset theory and research to a young clinical population with IBD.

## 1. Introduction

Inflammatory bowel diseases (IBD), including Crohn’s disease and ulcerative colitis, are chronic inflammatory conditions of the gastrointestinal tract characterized by a relapsing and remitting course [1,2]. Management often requires long-term pharmacologic treatment, routine monitoring, and sustained engagement in health behaviors [3,4,5]. Pediatric IBD poses distinct challenges compared to adult-onset disease, including more aggressive disease that may impact growth and development [6,7,8]. Pediatric IBD is also associated with significant psychosocial burden [9,10], as it involves youth managing a chronic illness during a developmental period marked by heightened biological and psychosocial change.

Adolescents and young adults (AYAs) with IBD experience elevated rates of stress, anxiety, depression and lower quality of life [10,11,12,13]. Adolescence also represents a high-risk period for nonadherence and suboptimal disease self-management, as responsibility for health behaviors shifts from caregivers to youth amid increasing academic, social, and developmental demands [14]. Psychological distress further undermines engagement in self-care, adherence, and self-efficacy in individuals with IBD [15,16,17]. Effective IBD management requires sustained motivation, persistence, and self-regulation [18,19,20], capacities that may be particularly challenging during adolescence and young adulthood. Consequently, IBD outcomes in AYAs are determined by the interplay of treatment efficacy, psychological functioning, and sustained adaptive self-management [21].

Emerging research suggests that positive psychological factors facilitate improved adjustment and health outcomes among AYAs with IBD [22,23]. For example, resilience has been associated with lower psychological distress and higher quality of life [24,25]. Additional psychological constructs such as self-efficacy and perceived control have been linked to improved IBD management and adherence behaviors [26,27], underscoring the importance of psychological processes in shaping IBD outcomes [28,29,30]. Of note, recent research emphasizes the role of positive, growth mindsets—beliefs that personal attributes are malleable and can change with effort—in shaping behaviors and wellness [26,31].

Mindset theory, developed by Carol Dweck and colleagues, conceptualizes individuals’ beliefs about the malleability of personal attributes (e.g., intelligence, abilities, skills) along a continuum from fixed (i.e., traits are static) to growth (i.e., traits can change with effort and experience) [32]. Growth mindsets are associated with greater resilience, persistence, and more adaptive responses to challenge and failure [31,33]. Extending this work to health, a growth health mindset reflects the belief that health is malleable and responsive to behavior and effort, whereas a fixed health mindset reflects the belief that health is largely predetermined and unchangeable [31,34,35,36]. Research in healthy and clinical populations shows that a growth health mindset is associated with engagement in health-promoting behaviors (e.g., physical activity, healthy diet, stress management, adherence to medical recommendations) and higher quality of life [31,34,36,37]. In contrast, a fixed health mindset is linked to poorer psychosocial outcomes [34].

A growth health mindset may be especially relevant for AYAs with IBD navigating unpredictable disease courses, symptom flares, and treatment changes alongside developmental demands. Although the shift to self-managed care is a known period of vulnerability, psychological frameworks used to interpret these challenges remain underexplored. In this context, a fixed health mindset may lead AYAs to interpret IBD challenges as evidence of permanent health decline or personal failure, fostering disengagement in care. Conversely, a growth health mindset may function as a psychological buffer, framing IBD setbacks as challenges to be managed through engagement in care. Research has not examined health mindset in AYAs with IBD, highlighting a gap in the IBD literature.

The primary aim of this study was to assess the internal reliability of the Health Mindset Scale in AYAs with IBD. The secondary aim was to examine associations between health mindset and important IBD-related outcomes, including psychosocial (depressive symptoms, peer relationships, global health) and physical (pain interference, fatigue, disease activity) outcomes. It was hypothesized that the Health Mindset Scale would demonstrate good internal reliability and that a growth health mindset would be associated with lower levels of depressive symptoms, pain interference, and fatigue and higher levels of peer relationships and global health in AYAs with IBD.

## 2. Materials and Methods

### 2.1. Participants

This study included AYAs living with IBD. Eligibility criteria included: (1) a self-reported diagnosis of ulcerative colitis, Crohn’s disease, or IBD unclassified; (2) age between 15 and 25 years; and (3) English proficiency. Participants were recruited between February 2020 and October 2021 through the Stanford Medicine Children’s Health Center for IBD and Celiac Disease and ImproveCareNow (ICN), an international pediatric IBD research and quality improvement network [38].

### 2.2. Procedure

This cross-sectional study involved completing a one-time online survey. Eligible patients receiving care at Stanford Medicine Children’s Health Center for IBD and Celiac Disease were introduced to the study during their appointments. To ensure geographical diversity, recruitment was extended through ICN. During the study period, ICN posted study information on its research opportunities webpage, allowing interested youth and families from across the network to participate.

For participants 17 years old or younger, caregivers provided electronic informed consent and participants provided electronic assent. Participants aged 18 years and older provided electronic informed consent. Following consent, participants completed the online survey. All assent, consent, and survey data were collected and managed in Stanford REDCap (Research Electronic Data Capture), a secure, web-based system designed to support clinical and research data management. All study procedures were reviewed and approved by the Stanford University Institutional Review Board (approval no. 50964).

### 2.3. Measures

#### 2.3.1. Demographics

Participants completed a self-report questionnaire assessing demographic characteristics (e.g., age, gender, race, ethnicity) and medical information [e.g., IBD diagnosis, age of diagnosis, co-occurring conditions (e.g., celiac disease, rheumatological disorders, autoimmune thyroid disease, irritable bowel syndrome, pain conditions), and mental health diagnoses (e.g., anxiety, depression, ADHD, other specified conditions)]. Additionally, the questionnaire assessed treatment-related factors, including current IBD medical treatments (see Supplementary Material S1).

#### 2.3.2. Health Mindset

Health mindset was assessed using the Health Mindset Scale, a three-item measure adapted from Dweck’s original mindset framework to assess beliefs about the malleability of health [34,39]. Items assessed the extent to which individuals believe health is fixed versus changeable (e.g., “Your body has a certain amount of health, and you really can’t do much to change it”). Responses were rated on a 6-point Likert scale ranging from 1 (strongly agree) to 6 (strongly disagree) and summed to create a total score. Higher scores indicated a belief that health can change over time, which is associated with a growth mindset of health. Lower scores indicated a belief that health cannot change over time, which is associated with a fixed mindset of health.

The Health Mindset Scale demonstrated good psychometric properties in a healthy high school student population, including strong correlations among items (r = 0.46–0.51) and good internal reliability (α = 0.73) [40]. Additional work has supported that the measure has good convergent validity, such as associations between health mindset and beliefs about genetic versus behavioral influences on health [40]. The scale has also demonstrated good internal reliability in pediatric and young adult healthy and clinical populations, such as chronic headache, postoperative recovery, and idiopathic scoliosis [26,39,40,41].

#### 2.3.3. Study Outcomes

Participants completed a series of Patient-Reported Outcomes Measurement Information System (PROMIS) measures assessing psychosocial health (i.e., depressive symptoms, peer relationships), physical health (i.e., pain interference, fatigue) and global health outcomes [41,42]. The PROMIS Pediatric Profile short forms were selected to reduce participant burden while allowing for efficient assessment of multiple health domains relevant to AYAs with IBD. These profile measures consist of validated domain-specific subscales derived from larger PROMIS item banks and have demonstrated strong reliability, validity, and sensitivity to change in pediatric populations, including youth with Crohn’s disease and ulcerative colitis [43,44].

All PROMIS items were rated on a 5-point Likert scale (e.g., never to always; excellent to poor). Most measures asked participants to reflect on their experiences over the past 7 days, whereas the global health measure asked about their general health and well-being. Raw PROMIS scores were entered into the HealthMeasures Scoring Service (https://www.assessmentcenter.net/ac_scoringservice; accessed on 2 October 2025), which converts responses into standardized T-scores. T-scores are normed to reference populations that include general population and clinical samples (mean = 50), with higher scores indicating a greater level of the construct assessed.

Depressive symptoms were measured through a four-item subscale from the PROMIS Pediatric Profile v2.0—Profile-25 [42,45]. Peer relationships were measured using a four-item subscale from the PROMIS Pediatric Profile v2.0—Profile-25 [41]. Global health was measured through seven items from the PROMIS Pediatric Scale v1.0—Global Health 7 [46]. Pain interference was measured using a four-item subscale from the PROMIS Pediatric Profile v2.0—Profile-25 [47], reflecting the extent to which pain disrupts physical, emotional, and social functioning. Fatigue was assessed using a four-item subscale from the PROMIS Pediatric Profile v2.0—Profile-25 [47].

Crohn’s disease activity was assessed using the short Crohn’s Disease Activity Index (sCDAI) [48]. Unlike the physician-rated PCDAI, the sCDAI is a patient-reported measure of disease activity and has been used in prior IBD research for self-assessment [49]. The sCDAI was originally developed using seven consecutive daily ratings of stool frequency, abdominal pain, and general well-being. Given the cross-sectional design of the present study, participants provided ratings for a single day, which were multiplied by seven to approximate weekly symptom activity, consistent with prior adaptations of the measure [48]. Scores on the adapted sCDAI range from a minimum of 44 to greater than 450, with established cutoffs used to characterize disease activity (remission: <150; mild: 150–219; moderate: 220–450; severe: >450).

Ulcerative colitis disease activity was assessed using the Pediatric Ulcerative Colitis Activity Index (PUCAI) via patient report [50]. Although the PUCAI is traditionally clinician-administered, prior research supports its use as a patient-reported measure in pediatric IBD populations, especially given the limited availability of validated self-report disease activity indices [43,51]. The PUCAI is a validated, noninvasive measure of disease activity that assesses six clinical domains, including abdominal pain, rectal bleeding, stool consistency, number of stools per 24 h, nocturnal stools, and activity level. Of note, AYAs who endorsed IBD unclassified also completed the PUCAI measure. Scores range from 0 to 85, with established cutoffs used to characterize disease activity (remission: <10; mild: 10–34; moderate: 35–64; severe: ≥65).

### 2.4. Statistical Analyses

#### 2.4.1. Descriptive Statistics

Descriptive statistics were calculated for demographic variables (i.e., age, sex, race/ethnicity) and medical variables (i.e., IBD diagnosis, years from diagnosis) using means and standard deviations or frequencies. To address potential non-normality in scores, means, standard deviations (SDs), medians, and interquartile ranges (IQRs) were calculated for all study measures, including health mindset, depressive symptoms, peer relationships, global health, pain interference, fatigue, and disease activity. Normality of the distributions was formally assessed using the Shapiro–Wilk test and visual inspection of Q-Q plots.

#### 2.4.2. Internal Consistency of Health Mindset

To address the primary aim, internal reliability of the Health Mindset Scale was evaluated using Cronbach’s alpha.

#### 2.4.3. Inferential Statistics

To address the secondary aim, bivariate associations between health mindset and physical and psychosocial outcomes were examined. Pearson’s r was used for variables meeting normality assumptions, and Spearman’s rho was used for variables with non-normal distributions. Hierarchical linear regression (HLR) analyses were then performed to evaluate the unique contribution of health mindset to each outcome after controlling for age and sex. HLR was selected to assess the incremental variance explained by health mindset beyond demographic covariates. These demographic factors (i.e., age, sex) were included a priori given evidence of association with outcomes. In each model, age and sex were entered in Step 1, followed by health mindset in Step 2. Disease activity was modeled as an outcome to explore associations between health mindset and subjective symptom burden, consistent with biopsychosocial frameworks in other pediatric chronic illness populations [52]. For each model, overall model statistics, change in explained variance, Cohen’s *f*^2^, and regression coefficients for all predictors are reported. Cohen’s *f*^2^ values of 0.02, 0.15, and 0.35 were utilized to represent small, medium, and large effect sizes [53].

## 3. Results

### 3.1. Study Population

A total of 101 AYAs with IBD participated in the study. Demographic and medical characteristics are summarized in Table 1. The mean age of the participants was 18.4 years (SD = 2.7), and the mean IBD duration was 4.5 years (SD = 4.0). Of the sample, 54.5% had ulcerative colitis or IBD unclassified, 63.4% were female, 60.4% were White, and 85.1% were non-Hispanic. The majority of participants (65.3%) had a disease activity score classified as in remission. Descriptive statistics for study outcomes are displayed in Table 1.

### 3.2. Internal Reliability Analysis

A Cronbach’s alpha value of 0.90 was obtained for the Health Mindset Scale. This indicates that the scale demonstrated excellent internal consistency for AYAs with IBD.

### 3.3. Correlational Analyses

Correlation analyses demonstrated that health mindset was significantly associated with peer relationships (*ρ* = 0.20, *p* = 0.04), pain interference (*ρ* = −0.25, *p* = 0.01), and CDAI (*ρ* = −0.36, *p* = 0.01). Participants with a growth health mindset reported higher quality peer relationships, less pain interference, and less severe Crohn’s disease activity. Health mindset was not found to be significantly associated with depressive symptoms (*ρ* = −0.02, *p* = 0.88), global health (*r* = 0.19, *p* = 0.05), fatigue (*ρ* = 0.04, *p* = 0.68), or PUCAI (*ρ* = −0.01, *p* = 0.91).

### 3.4. Hierarchical Linear Regression Analyses

HLR analyses examining the unique contribution of health mindset to IBD outcomes, after controlling for age and sex, are summarized in Table 2.

Depressive symptoms. The overall model examining depressive symptoms was significant (*F*(3, 97) = 4.81; *p* = 0.004). Age and sex significantly contributed 12.7% of the variance in depressive symptoms (*F*(2, 98) = 7.15; *p* = 0.001). Health mindset did not significantly account for additional variance (Δ*R*^2^ = 0.002; *F*(1, 97) = 0.24; *p* = 0.63). Health mindset was not a significant independent predictor of depressive symptoms (*β* = −0.05, *p* = 0.63; 95% CI: −0.55 to 0.33; Cohen’s *f*^2^ = 0.002).

Peer relationships. The overall model examining quality of peer relationships was significant (*F*(3, 97) = 3.28; *p* = 0.024). Age and sex did not significantly contribute to the variance in peer relationship quality (*R*^2^ = 0.06; *F*(2, 98) = 3.05; *p* = 0.05). Health mindset did not significantly account for additional variance (Δ*R*^2^ = 0.03; *F*(1, 97) = 3.58, *p* = 0.06). Health mindset was not a significant independent predictor of peer relationships (β = 0.18, *p* = 0.06, 95% CI: −0.02 to 0.82); however, it demonstrated a small effect size (Cohen’s *f*^2^ = 0.04).

Global health. The overall model examining global health was significant (*F*(3, 97) = 5.80; *p* = 0.001). Age and sex significantly contributed 10.9% to the variance in global health (*F*(2, 98) = 6.00; *p* = 0.003). Health mindset significantly accounted for an additional 4.3% of variance (*F*(1, 97) = 4.92; *p* = 0.03). Health mindset was a significant independent predictor of global health with a small effect (*β* = 0.21, *p* = 0.03; 95% CI: 0.04 to 0.79; Cohen’s *f*^2^ = 0.05). Participants who reported a growth health mindset reported greater global health.

Pain interference. The overall model examining pain interference was significant (*F*(3, 97) = 2.94; *p* = 0.037). Age and sex did not significantly contribute to the variance in pain interference (*R*^2^ = 0.02; *F*(2, 98) = 0.95; *p* = 0.39). Health mindset significantly accounted for an additional 6.4% of variance (*F*(1, 97) = 6.82; *p* = 0.01). Health mindset was a significant independent predictor of pain interference with a small effect (*β* = −0.26, *p* = 0.01; 95% CI: −0.58 to −0.08; Cohen’s *f*^2^ = 0.07). Participants who reported a growth health mindset experienced less pain interference.

Fatigue. The overall model examining fatigue was not significant (*F*(3, 97) = 1.90; *p* = 0.134). Age and sex did not significantly contribute to the variance in fatigue (*R*^2^ = 0.05; *F*(2, 98) = 2.74; *p* = 0.07). Health mindset did not significantly account for additional variance (∆*R*^2^ = 0.003; *F*(1, 97) = 0.28; *p* = 0.60). Health mindset was not a significant independent predictor of fatigue (*β* = 0.05, *p* = 0.60; 95% CI: −0.26 to 0.46; Cohen’s *f*^2^ = 0.003).

Disease activity. Among participants with Crohn’s disease, the overall model predicting disease activity was significant (*F*(3, 42) = 4.00; *p* = 0.01). Age and sex did not significantly account for the variance in disease activity (*R*^2^ = 0.02; *F*(2, 43) = 0.49; *p* = 0.62). Health mindset significantly accounted for an additional 20% of variance (*F*(1, 42) = 10.82; *p* = 0.002). Health mindset was a significant independent predictor of Crohn’s disease activity with a medium effect (*β* = −0.46, *p* = 0.002; 95% CI: −9.67 to −2.32; Cohen’s *f*^2^ = 0.26). Participants with Crohn’s disease who reported a growth health mindset reported less severe disease activity.

In contrast, the overall model examining ulcerative colitis disease activity was not significant (*F*(3, 51) = 0.24; *p* = 0.866). Age and sex did not significantly contribute to the variance in disease activity (*R*^2^ = 0.01; *F*(2, 52) = 0.30; *p* = 0.74). Health mindset did not significantly account for additional variance (∆*R*^2^ = 0.003; *F*(1, 51) = 0.14; *p* = 0.71). Health mindset was not a significant independent predictor of ulcerative colitis disease activity (*β* = −0.05, *p* = 0.71; 95% CI: −1.30 to 0.89; Cohen’s *f*^2^ = 0.003).

## 4. Discussion

Health mindset, conceptualized as beliefs about whether one’s health is relatively fixed and predetermined or malleable and responsive to behaviors, was reliably assessed in AYAs with IBD. In this cross-sectional study, health mindset demonstrated meaningful associations with several key IBD outcomes. A growth health mindset was associated with less pain interference, lower Crohn’s disease activity, and improved global health among AYAs with IBD. In contrast, health mindset was not significantly associated with depressive symptoms, peer relationship quality, fatigue, or ulcerative colitis disease activity. Collectively, these findings suggest that beliefs about the malleability of health may influence how AYAs interpret, experience, and manage the day-to-day impact of living with IBD.

Notably, health mindset was significantly associated with pain interference. AYAs with a growth health mindset reported less pain-related disruption, suggesting that health mindset may shape how AYAs interpret and respond to IBD symptoms like pain. Viewing pain as manageable and responsive to effort, rather than as uncontrollable or indicative of permanent decline, may support persistence in valued activities despite discomfort [54]. While this finding had a small effect, it may be clinically meaningful given how psychosocial factors influence pain experiences. This aligns with recent IBD research highlighting the impact of positive psychological processes (e.g., self-efficacy, optimism, positive affect) on improved pain outcomes in children and adults with IBD [22,55]. Research in pediatric pain and chronic illness populations has also highlighted the role of psychological factors (e.g., self-efficacy, pain acceptance) in promoting functioning in the context of pain [56,57,58,59]. Moreover, these findings are consistent with the literature showing that growth mindsets are associated with adaptive pain outcomes across clinical populations [34,60].

Additionally, health mindset was significantly associated with Crohn’s disease activity, with those endorsing a growth mindset reporting lower disease activity. This moderate effect suggests that health mindset may be a relevant clinical factor. This finding also suggests that a growth health mindset may impact engagement in IBD management and, in turn, disease activity. AYAs who view their health as malleable may be more likely to persist in self-management behaviors (e.g., treatment adherence), supporting improved disease activity. AYAs who think that their health is fixed may be more likely to disengage in care. Although IBD self-management was not directly assessed, this interpretation aligns with research linking a growth health mindset to greater motivation and treatment engagement [35]. Future longitudinal research should examine whether health mindset moderates the relationship between IBD self-management and disease activity over time. Lastly, the relationship between health mindset and disease activity supports the literature linking psychological factors with improvements in inflammation and disease markers [61,62,63].

While the observed cross-sectional association between health mindset and Crohn’s disease activity is intriguing, it must be interpreted with caution considering limitations. Disease activity was assessed using self-report measures and was only significant among AYAs with Crohn’s disease, not ulcerative colitis. This raises questions about whether this result reflects differences in disease-specific processes or measurement-related factors. Health mindset may also influence the ‘appraisal’ of active disease, where an individual with a growth health mindset views fluctuations in their IBD symptoms as manageable rather than as signs of decline, influencing their reporting of disease activity. Of note, most AYAs were in remission or had mild disease, limiting generalizability to those with more severe disease. It is possible that variations in disease status influence AYAs’ health mindset. Individuals with treatment-refractory disease may perceive their IBD as fixed due to poor intervention response, whereas those with milder disease may more readily adopt a growth mindset given more positive clinical outcomes. Future research should prioritize longitudinal designs and investigate the relationship between health mindset and disease activity using multimethod assessments, including provider (e.g., physician global assessment) and biological (e.g., calprotectin stool test) markers of disease.

The study findings further demonstrated that AYAs who endorsed a growth health mindset reported improved global health. Global health is a clinically meaningful outcome, reflecting integrated physical, emotional, and social well-being, and has been linked to important outcomes in pediatric IBD, including improved treatment adherence and psychosocial functioning [64]. From a biopsychosocial perspective, having a growth health mindset may shape how individuals interpret and respond to IBD symptoms and challenges, thereby influencing functioning and wellbeing across physical, emotional, and social domains. The observed association between health mindset and pain interference supports this hypothesis, suggesting that adaptive health beliefs may contribute to better physical wellbeing despite ongoing symptoms. That stated, the association between health mindset and global health was small in magnitude, suggesting that it is likely one of multiple interacting factors influencing overall well-being.

While health mindset was not significantly associated with peer relationship quality, the confidence interval and small effect size suggests a potential signal that may become more pronounced in larger samples. These findings indicate that a growth health mindset may support relationship quality and overall social wellbeing during adolescence and young adulthood, a developmental period where peer belonging is central. In contrast, health mindset was not related to depression symptoms. This finding diverges from the past literature linking growth mindsets to improved psychological functioning [65,66]. One explanation for this result is that the sample reported sub-clinical depression symptoms comparable to the general population. Greater variability in psychological distress may be necessary to detect associations. While additional research is needed, the results suggest that a growth health mindset may support AYAs with the holistic experience of living with a chronic illness.

Despite these promising preliminary results, several limitations warrant consideration. First, the cross-sectional study design limits inferences about causal directionality between health mindset and IBD outcomes. It is unclear whether a growth health mindset facilitates improved IBD outcomes or if improved health outcomes support individuals in adopting a more malleable view of their health. Longitudinal research is required to determine if health mindsets serve as a precursor to improved clinical outcomes or a psychological response to stable health outcomes. Additionally, reliance on self-report measures may introduce reporting bias, and specific IBD self-management behaviors such as treatment adherence were not directly assessed. Lastly, as this study used an anonymous survey, there is always a possibility of fraudulent participants. This risk was reduced as recruitment efforts were focused on one IBD clinic and IBD organization, and there were no participation incentives.

Future research should build on these findings using longitudinal and mechanistic designs to clarify temporal pathways linking health mindset to disease activity and global health, as well as the role that self-management behaviors may play in this relationship. Examining health mindset across more clinically diverse IBD subpopulations, including AYAs with varying levels of disease and psychological distress, may further clarify when and for whom health mindset is most influential. Additionally, it will be important to explore health mindset as a target for IBD intervention development. Although no published studies to date have developed protocols targeting health mindset in IBD, a large body of social psychology research has shown that mindsets are modifiable [31,32,33,66]. Crum and colleagues demonstrated that brief interventions promoting a growth-oriented mindset improve health outcomes, including reduced physiological stress, more adaptive coping, and increased well-being across clinical and non-clinical samples [33,67,68].

Extending these interventions to AYAs with IBD may provide a novel pathway for improving health outcomes. Specifically, interventions that help AYAs see their health as malleable versus fixed may impact how they respond to health challenges, engage in self-management behaviors, and participate in valued activities. In line with the study findings, developing a growth health mindset has the potential to influence daily functioning and wellbeing among AYAs with IBD. Cultivating a growth health mindset during a developmental period marked by significant transition may bolster long-term IBD health outcomes and resilience.

## 5. Conclusions

This novel study provides preliminary evidence that having a growth health mindset is associated with clinically meaningful outcomes in AYAs with IBD, including improved pain interference, Crohn’s disease activity, and global health. The findings highlight that health mindset may be an important psychological construct in chronic illness adjustment and management. Research employing longitudinal, mechanistic, and interventional designs may inform the development of novel, scalable strategies that support the cultivation of a growth health mindset and improved IBD outcomes among AYAs with IBD.

## Figures and Tables

**Table 1:** Demographics (*n* = 101).

Variable	Mean (SD)	Median (IQR)	*n* (%)
Age	18.36 (2.73)	18.00 (16.00, 20.00)	
Years since diagnosis	4.53 (4.01)	3.00 (2.00, 7.00)	
Sex			
Female			64 (63.4)
Male			37 (36.6)
Race/Ethnicity			
White, Non-Hispanic			61 (60.4)
Black, Non-Hispanic			1 (1.0)
Hispanic or Latino			15 (14.9)
Native American or Alaskan Native			1 (1.0)
Asian or Pacific Islander			18 (17.8)
Other			5 (5.0)
IBD Diagnosis			
Crohn’s disease			46 (45.5)
Ulcerative colitis			52 (51.5)
IBD unclassified			3 (3.0)
Measures			
Health Mindset Scale	11.67 (4.60)	12.00 (8.00–15.00)	
Depressive symptoms	51.33 (10.78)	51.10 (40.45–59.80)	
Peer relationships	51.12 (9.97)	51.90 (45.05–61.10)	
Global health	43.81 (9.18)	43.30 (37.35–49.10)	
Pain interference	47.09 (5.95)	42.60 (42.60–50.30)	
Fatigue	50.22 (8.42)	52.90 (40.00–59.10)	
Crohn’s Disease Activity Index	122.52 (62.54)	128.00 (79.00–142.00)	
Pediatric Ulcerative Colitis Activity Index	15.81 (17.18)	10.00 (5.00–15.00)	

**Table 2:** Hierarchical linear regression analyses of health mindset and IBD outcomes ^a^.

Step and Variables		Statistics by Step		Statistics by Variable	
		*R* ^2^	Δ*R*^2^	Final Std. *β*	95% CI
Outcome	Depressive symptoms				
1.	Age			0.24 *	0.19, 1.67
	Sex	0.127	0.127 **	0.27 **	1.83, 10.23
2.	Health mindset	0.129	0.002	−0.05	−0.55, 0.33
Outcome	Peer relationships				
1.	Age			−0.19	−1.40, 0.01
	Sex	0.059	0.059	0.13	−1.34, 6.59
2.	Health mindset	0.092	0.033	0.18	−0.02, 0.82
Outcome	Global health				
1.	Age			−0.23 *	−1.38, −0.13
	Sex	0.109	0.109 **	−0.26 **	−8.36, −1.30
2.	Health mindset	0.152	0.043 *	0.21 *	0.04, 0.78
Outcome	Pain interference				
1.	Age			−0.12	−0.67, 0.17
	Sex	0.019	0.019	0.11	−1.09, 3.67
2.	Health mindset	0.083	0.064 *	−0.26 *	−0.58, −0.08
Outcome	Fatigue				
1.	Age			0.18	−0.05, 1.15
	Sex	0.053	0.053	0.14	−0.92, 5.91
2.	Health mindset	0.056	0.003	0.05	−0.26, 0.46
Outcome	CDAI				
1.	Age			−0.14	−9.34, 3.08
	Sex	0.022	0.022	0.23	−7.35, 68.21
2.	Health mindset	0.222	0.200 **	−0.46 **	−9.67, −2.32
Outcome	PUCAI				
1.	Age			−0.05	−2.08, 1.49
	Sex	0.011	0.011	0.09	−6.43, 12.87
2.	Health mindset	0.014	0.003	−0.05	−1.30, 0.89

^a^ All standardized beta coefficients are from the final step in the HLR analyses. * *p* < 0.05, ** *p* < 0.01.

## Data Availability

The data presented in this study are available on request from the corresponding author. The data are not publicly available due to privacy.

## Supplementary Materials

The following supporting information can be downloaded at: https://www.mdpi.com/article/10.3390/children13050658/s1.

## Abbreviations

The following abbreviations are used in this manuscript:

AYA	Adolescent and Young Adult
IBD	Inflammatory Bowel Disease
ICN	ImproveCareNow
REDCap	Research Electronic Data Capture
PROMIS	Patient-Reported Outcomes Measurement Information System
sCDAI	Short Crohn’s Disease Activity Index
PUCAI	Pediatric Ulcerative Colitis Activity Index
HLR	Hierarchical Linear Regression
PGA	Physician Global Assessment
SD	Standard Deviation
CI	Confidence Interval
