# Supplementary material for: Health Mindset in Pediatric Inflammatory Bowel Disease: Exploring the Relationship Between Health Mindset and Key Physical and Psychosocial Outcomes
Source: Children (Basel). 2026 May 8;13(5):658. doi: 10.3390/children13050658 (PMC13204204; doi:10.3390/children13050658)
Supplement: Supplementary file 1 [file children-13-00658-s001.zip › Supplementary Material S1.pdf]

Demographic Questionnaire

1. Age:_____________

2. Sex:
   Male _______
   Female_______
   Other________

3. Race/Ethnicity
   Black, non-Hispanic_________
   Hispanic or Latino__________
   Native American or Alaskan Native_________
   Asian or Pacific Islander___________
   White, non-Hispanic__________
   Other____________ (Please Specify:_____________________________)

4. IBD diagnosis:
   Crohn's____________
   Ulcerative colitis_____________
   Indeterminate colitis______________

5. Age at IBD diagnosis: _____________

6. Current IBD medications:
   - Biologics (infliximab-Remicade, adalimumab-Humira, certolizumab-Cimzia, vedolizumab-Entyvio, ustekinumab-Stelara):
     Yes___ No____
   - Immunomodulator (methotrexate, azathioprine, 6-mercaptopurine): Yes__ No_
   - Oral corticosteroids (prednisone, prednisolone, uceris, budesonide):
     Yes___ No____
   - 5-ASA (sulphasalazine-Salazopyrin; mesalazine-Asacol, Ipocol, Octasa, Pentasa, Salofalk, Mezavant; Olsalazine-Dipentum; balsalazide-Colazide):
     Yes _____ No____

7. Have you had an IBD-related surgery? Yes_____ No______ Unknown______

   If yes, how many surgeries have you had? 1____ 2____ 3 or more____

8. Other medical/mental health diagnoses:
   Celiac disease: Yes___ No____
   Rheumatologic disorder: Yes___ No____
   Autoimmune thyroid disease: Yes___ No____
   Irritable bowel syndrome: Yes___ No____
   Pain Condition: Yes___ No____ (If yes, please specify:________________)
   Mental Health Diagnosis: Yes___ No____

Anxiety: Yes___ No____
Depression: Yes___ No____
ADHD: Yes___ No____
Other: Yes___ No____ (Please specify:_________________________)

9. Are you currently receiving mental health treatment? Yes_____ No_______

If yes, type of treatment:_________________________________

10. In the past 6 months, how many times have you been seen in an urgent care center, emergency room department, and/or required hospitalization?
0____
1____
2____
3 or more____
4 >1 but don't know how many times

What was the reason? Please check all that apply.
Infection_____
Flare______
Surgery______
Mental health_____
Other______ (Please specify :____________________________________)
Unknown________

HEALTH MINDSET SCALE

ANSWER THE FOLLOWING QUESTIONS. FOR EACH ITEM WE WOULD LIKE YOU TO CIRCLE THE NUMBER THAT REPRESENTS THE EXTENT TO WHICH YOU AGREE OR DISAGREE WITH THAT STATEMENT. THE MORE YOU DISAGREE, THE HIGHER THE NUMBER YOU CIRCLE.

1. YOUR BODY HAS A CERTAIN AMOUNT OF HEALTH, AND YOU REALLY CAN'T DO MUCH TO CHANGE IT.

1	2	3	4	5	6
STRONGLY AGREE					STRONGLY DISAGREE

2. YOUR HEALTH IS SOMETHING ABOUT YOU THAT YOU CAN'T CHANGE VERY MUCH.

1	2	3	4	5	6
STRONGLY AGREE					STRONGLY DISAGREE

3. YOU CAN TRY TO MAKE YOURSELF FEEL BETTER, BUT YOU CAN'T REALLY CHANGE YOUR BASIC HEALTH.

1	2	3	4	5	6
STRONGLY AGREE					STRONGLY DISAGREE

Pediatric Profile-25

Please respond to each question or statement by marking one box per row.

	**Physical Function Mobility**<br>**In the past 7 days…**	**With no trouble**	**With a little trouble**	**With some trouble**	**With a lot of trouble**	**Not able to do**
236R1r	I could do sports and exercise that other kids my age could do................................	☐ 5	☐ 4	☐ 3	☐ 2	☐ 1
4124R1r	I could get up from the floor	☐ 5	☐ 4	☐ 3	☐ 2	☐ 1
2707R2r	I could walk up stairs without holding on to anything...	☐ 5	☐ 4	☐ 3	☐ 2	☐ 1
6023R1r	I have been physically able to do the activities I enjoy most	☐ 5	☐ 4	☐ 3	☐ 2	☐ 1
	**Anxiety**<br>**In the past 7 days…**	**Never**	**Almost Never**	**Sometimes**	**Often**	**Almost Always**
2220R2r	I felt like something awful might happen..	☐ 1	☐ 2	☐ 3	☐ 4	☐ 5
713R1r	I felt nervous..	☐ 1	☐ 2	☐ 3	☐ 4	☐ 5
6044R1r	I felt worried..	☐ 1	☐ 2	☐ 3	☐ 4	☐ 5
3458bR1r	I worried when I was at home	☐ 1	☐ 2	☐ 3	☐ 4	☐ 5
	**Depressive Symptoms**<br>**In the past 7 days...**	**Never**	**Almost Never**	**Sometimes**	**Often**	**Almost Always**
6041R1r	I felt everything in my life went wrong......	☐ 1	☐ 2	☐ 3	☐ 4	☐ 5
711R1r	I felt lonely	☐ 1	☐ 2	☐ 3	☐ 4	☐ 5
228R1r	I felt sad..	☐ 1	☐ 2	☐ 3	☐ 4	☐ 5
3952aR2r	It was hard for me to have fun...................	☐ 1	☐ 2	☐ 3	☐ 4	☐ 5
	**Fatigue**<br>**In the past 7 days…**	**Never**	**Almost Never**	**Sometimes**	**Often**	**Almost Always**
4238aR2r	Being tired made it hard for me to keep up with my schoolwork	☐ 1	☐ 2	☐ 3	☐ 4	☐ 5
2876R1r	I got tired easily..	☐ 1	☐ 2	☐ 3	☐ 4	☐ 5

© 2010-2016 PROMIS Health Organization and PROMIS Cooperative Group

	**Fatigue**<br>**In the past 7 days…**	**Never**	**Almost Never**	**Sometimes**	**Often**	**Almost Always**
4241R2r	I was too tired to do sports or exercise.......	☐ 1	☐ 2	☐ 3	☐ 4	☐ 5
4198R1r	I was too tired to enjoy the things I like to do..	☐ 1	☐ 2	☐ 3	☐ 4	☐ 5
	**Peer Relationships**<br>**In the past 7 days…**	**Never**	**Almost Never**	**Sometimes**	**Often**	**Almost Always**
5018R1r	I felt accepted by other kids my age..........	☐ 1	☐ 2	☐ 3	☐ 4	☐ 5
5058R1r	I was able to count on my friends	☐ 1	☐ 2	☐ 3	☐ 4	☐ 5
5062R1r	My friends and I helped each other out......	☐ 1	☐ 2	☐ 3	☐ 4	☐ 5
233R2r	Other kids wanted to be my friend	☐ 1	☐ 2	☐ 3	☐ 4	☐ 5
	**Pain Interference**<br>**In the past 7 days…**	**Never**	**Almost Never**	**Sometimes**	**Often**	**Almost Always**
3793R1r	I had trouble sleeping when I had pain.......	☐ 1	☐ 2	☐ 3	☐ 4	☐ 5
9004r	It was hard for me to pay attention when I had pain	☐ 1	☐ 2	☐ 3	☐ 4	☐ 5
2045R1r	It was hard for me to run when I had pain...	☐ 1	☐ 2	☐ 3	☐ 4	☐ 5
2049R1r	It was hard for me to walk one block when I had pain..	☐ 1	☐ 2	☐ 3	☐ 4	☐ 5

	**Pain Intensity**<br>**In the past 7 days…**											
9033R1r	How bad was your pain on average?...	☐ 0<br>**No pain**	☐ 1	☐ 2	☐ 3	☐ 4	☐ 5	☐ 6	☐ 7	☐ 8	☐ 9	☐ 10<br>**Worst pain you can think of**

© 2010-2016 PROMIS Health Organization and PROMIS Cooperative Group

Pediatric Global Health 7

Please respond to each question or statement by marking one box per row.

		Excellent	Very Good	Good	Fair	Poor
Global01R1	In general, would you say your health is:	☐ 5	☐ 4	☐ 3	☐ 2	☐ 1
Global02R1	In general, would you say your quality of life is:	☐ 5	☐ 4	☐ 3	☐ 2	☐ 1
Global03R1	In general, how would you rate your physical health?	☐ 5	☐ 4	☐ 3	☐ 2	☐ 1
Global04R1	In general, how would you rate your mental health, including your mood and your ability to think?	☐ 5	☐ 4	☐ 3	☐ 2	☐ 1

		Never	Rarely	Sometimes	Often	Always
PedGlobal2R1	How often do you feel really sad?	☐ 5	☐ 4	☐ 3	☐ 2	☐ 1

		Always	Often	Sometimes	Rarely	Never
PedGlobal5R1	How often do you have fun with friends?	☐ 5	☐ 4	☐ 3	☐ 2	☐ 1
PedGlobal6R1	How often do your parents listen to your ideas?	☐ 5	☐ 4	☐ 3	☐ 2	☐ 1

© 2010-2016 PROMIS Health Organization and PROMIS Cooperative Group

Short Crohn's Disease Activity Index (sCDAI)

Please answer the questions below if you've been diagnosed with Crohn's:

Over the past 7 days……

1) Daily abdominal pain rating:

   0=None
   1=Mild
   2=Moderate
   3=Severe

2) Number of liquid or soft stools each day:
   0
   1
   2
   3
   4
   5
   6 or more

3) General well-being:
   0=Generally well
   1=Slightly under par
   2=Poor
   3=Very poor
   4=Terrible

Pediatric Ulcerative Colitis Activity Index (PUCAI)

ITEM	POINTS
**1. Abdominal pain:**	
No pain	0
Pain can be ignored	5
Pain cannot be ignored	10
**2. Rectal bleeding**	
None	0
Small amount only, in less than 50% of stools	10
Small amount with most stools	20
Large amount (>50% of the stool content)	30
**3. Stool consistency of most stools**	
Formed	0
Partially formed	5
Completely unformed	10
**4. Number of stools per 24 hours**	
0-2	0
3-5	5
6-8	10
>8	15
**5. Nocturnal bowel movement (any diarrhea episode causing wakening)**	
No	0
Yes	10
**6. Activity level**	
No limitation of activity	0
Occasional limitation of activity	5
Severe restricted activity	10
SUM OF PUCAI (0-85)	
